# Identification of a mechanical signaling pathway activating type 1 fimbriae via a ZraSR two-component system in neonatal meningitis-associated *Escherichia coli*

**DOI:** 10.1080/21505594.2026.2721762

**Published:** 2026-09-11

**Authors:** Xiaoya Li, Jiake Wang, Zhengang Li, Xi Guo, Yuchen Wang, Xintong Chen, Yangyang Zheng, Qiyue Zhang, Ruiying Liu, Xuewei Sun, Bin Liu, Hao Sun

**Affiliations:** a National Key Laboratory of Intelligent Tracking and Forecasting for Infectious Diseases, TEDA Institute of Biological Sciences and Biotechnology, Nankai University, Tianjin, China; b The Key Laboratory of Molecular Microbiology and Technology, Ministry of Education, Nankai University, Tianjin, China; c Chinese Academy of Medical Sciences, State Key Laboratory of Experimental Hematology, Institute of Hematology & Blood Diseases Hospital, Tianjin, China; d Department of Critical Care Medicine, Children’s Hospital of Tianjin University/Tianjin Children’s Hospital, Tianjin, China; e Nankai International Advanced Research Institute, Nankai University, Shenzhen, China

**Keywords:** *Escherichia coli*, two-component system, type 1 fimbriae, host-pathogen interaction, meningitis, envelope stress response

## Abstract

*Escherichia coli* K1 (*E. coli* K1) meningitis develops after the bacteria cross the blood−brain barrier (BBB); however, the molecular mechanisms underlying this process remain incompletely understood. Adhesion to human brain microvascular endothelial cells (HBMECs), which constitute the BBB, is considered important in *E. coli* K1 bacterial penetration into the central nervous system. Here, we report a complete signal regulatory pathway in which the ZraSR two-component system (TCS) senses mechanical cues-specifically, physical forces and envelope perturbations generated by the initial attachment of *E. coli* K1 to host cells-and directly binds to *fimS*, the invertible promoter element of the *fim* operon (*fimAICDFGH*). This interaction promotes *fimS* inversion to the phase-ON orientation, thereby activating the expression of type 1 fimbriae, specifically the FimH adhesin. This activation enhances *E. coli* K1 invasion into HBMECs. Disruption of this signaling pathway severely attenuates the progression of meningitis and reduces the production of proinflammatory factors triggered by *E. coli* K1 infection *in vivo*. These findings suggest that components of this pathway may serve as potential targets for novel therapeutic strategies against *E. coli* K1 infection.

## Introduction

*Escherichia coli* carrying the K1 capsule (*E. coli* K1) is the most common cause of neonatal bacterial meningitis [1]. Despite advancements in antimicrobial treatment and supportive care, infection-associated mortality remains high, ranging from 5% to 30%, with nearly half of survivors developing long-term neurological complications [2–5].

The infection typically initiates via vertical transmission from mother to newborn, followed by bacterial colonization of mucosal surfaces – primarily in the upper respiratory or gastrointestinal tract [6–8]. After establishing colonization, *E. coli* K1 breaches the epithelial barrier, enters the bloodstream, and achieves a high degree of bacteremia [9,10]. This systemic dissemination is facilitated by the K1 polysaccharide capsule, a critical virulence factor that enables immune evasion by resisting complement-mediated killing [11]. Subsequently, *E. coli* K1 exploits additional virulence factors to adhere and invade human brain microvascular endothelial cells (HBMECs), which are the key structural and functional components of blood−brain barrier (BBB), ultimately resulting in bacterial meningitis [12].

*E. coli* K1 adhesion to and invasion of HBMECs is a prerequisite for bacterial penetration into the brain [13]. Research has established that type 1 fimbriae, particularly the FimH adhesin, serve dual functions in this process: not only do they mediate bacterial attachment to the endothelial cell surface, but they also initiate specific signaling cascades that facilitate cellular invasion [14]. FimH-CD48 interaction triggers a series of intracellular events, including elevated cytosolic free calcium levels and RhoA activation, which are known to be involved in the host cell actin cytoskeletal rearrangements and ultimately promote *E. coli* K1 internalization into HBMECs [14]. Deletion of *fimH* in *E. coli* K1 significantly attenuated bacterial adhesion to and invasion of HBMECs [15]. Notably, expression of type 1 fimbriae in wild-type *E. coli* K1 is regulated by phase variation in which each bacterium can alternate between fimbriated (phase-ON) and non-fimbriated (phase-OFF) states [10,15]. Previous study demonstrated that *E. coli* K1 associated with HBMECs are found to be predominantly type 1 fimbriae phase-ON bacteria [15], highlighting the crucial role of fimbrial phase variation in bacterial virulence.

The expression of type 1 fimbriae is regulated through phase variation mediated by *fimS*, a 314-bp invertible DNA element that controls transcription of the *fim* operon (*fimAICDFGH*) [16,17]. This genetic switch is controlled by two 9-bp inverted repeat sequences (IRR and IRL), which serve as recognition sites for DNA inversion [16,18]. When *fimS* is oriented in the phase-ON position, the *fim* operon (*fimAICDFGH*) is actively transcribed; however, inversion to the phase-OFF orientation abolishes expression [16]. Adjacent to *fimS* are *fimB* and *fimE*, genes encoding tyrosine recombinases that specifically bind to IRR and IRL to catalyze the site-specific DNA inversion responsible for phase variation [16]. Therefore, the optimal expression of the FimB and FimE recombinases is crucial for regulating type 1 fimbriae phase variation in response to multiple environmental conditions. Several factors including the stationary phase σ factor (RpoS) and response regulator OmpR have been implicated in this process [19,20]. Additionally, others demonstrated that both the integration host factor (IHF) and the leucine-responsive regulatory protein (Lrp) bind to *fimS* directly, promoting the phase-ON orientation by facilitating a recombination-proficient structure [21,22]. Nevertheless, the comprehensive regulatory network governing type 1 fimbriae expression during neonatal meningitis-causing *E. coli* (NMEC) infection remains unclear.

The two-component regulatory system (TCS) are hallmark regulatory machineries widely distributed in bacteria, consisting of an inner membrane histidine sensor kinase and a cytoplasmic response regulator. TCS regulates the expression of different sets of genes that enable bacteria to adapt their cellular process and virulence to a wide variety of environmental signals [23,24]. A common and critical environmental challenge faced by bacteria is disturbance to the cell envelope, which directly interfaces with the external milieu. Envelope integrity is essential for protecting cells from stressful and fluctuating conditions, and the ability to sense envelope damage, maintain cell wall structure, and repair assembly defects is vital for bacterial survival and pathogenesis [25]. Notably, many TCSs in pathogenic bacteria function as envelope stress responses (ESRs) that specifically detect cell envelope perturbation and rapidly control the genes expression to mount adaptive responses. For instance, the EnvZ-OmpR TCS in uropathogenic *E. coli* (UPEC) indirectly represses type 1 fimbriae expression by modulating inversion of the *fim* switch [20], promoting bacterial colonization under the high-osmolarity and acidic conditions of the bladder. QseC directly dephosphorylates QseB, thereby relieving the repression of type 1 fimbriae expression and facilitating UPEC colonization and intracellular bacterial community (IBC) formation [26]. In *E. coli* K1, the ZraSR TCS, a member of the envelope stress response network (ESR), is transcriptionally induced upon host cell attachment [27,28]. Given that related stress systems sense mechanical cues to activate virulence programs, the regulatory logic and mechanistic basis underlying ZraSR function remain largely undefined.

As the activation of ZraSR during HBMECs infection, we supposed that the TCS ZraSR, may enhancing *E. coli* K1 invasion of HBMECs and progression of meningitis upon sensing mechanical cues generated by initial host cell attachment. To test this hypothesis, we investigated the role of ZraSR in *E. coli* K1 adhesion to and invasion of HBMECs and its underlying regulation pathway. Our study revealed that the ZraSR TCS responds to the mechanical cue generated from initial host adherence and directly binds to *fimS*, which controls transcription of the *fim* operon. This interaction increases the expression of *fimH*, consequently enhancing *E. coli* K1 invasion of HBMECs. These findings enhance our understanding of the molecular mechanisms governing type 1 fimbriae-mediated adhesion and invasion in *E. coli* K1 pathogenesis.

## Materials and methods

### Ethics statement

All animal experiments were performed according to the ethical guidelines set forth in the Guide for the Care and Use of Laboratory Animals. The animal studies were approved by the Institutional Animal Care and Use Committee (IACUC) of Nankai University (Tianjin, China) and performed under protocol no. 2024-SYDWLL-000082 and adhered to the ARRIVE guidelines.

### Bacterial strains, plasmids and growth conditions

The wild-type (WT) strain used in this study was *Escherichia coli* RS218 (O18:K1:H7), a K1 capsule-expressing strain originally isolated from the cerebrospinal fluid of a newborn with meningitis [29]. This strain kindly provided by Dr. K.S. Kim (Johns Hopkins University, Baltimore, MD). All bacterial strains, plasmids, and primers used are listed in Supplementary Tables S1 and S2. Mutant strains were generated using the λ-red recombination system with plasmid pKD3 carrying a chloramphenicol resistance marker. Where necessary, the helper plasmid pCP20, which encodes FLP recombinase, was employed to excise the antibiotic resistance cassette. For the *zraR*-D56A point mutant, a synthetic donor DNA fragment containing the D56A mutation (GAT-GCT), flanked by upstream and downstream homology arms and a chloramphenicol resistance cassette, was introduced into the wild-type strain using the same λ-red recombination strategy. For genetic complementation, target genes together with their native promoters were cloned into the pTrc99A vector. To purify a 6×His-tagged ZraR protein, the *zraR* was cloned into the pET-28a expression vector and transferred into *E. coli* BL21 (DE3). For ChIP-qPCR, 3×FLAG-tagged version of *zraR* was constructed in pTrc99A and introduced into the Δ*zraSR* mutant strain. All constructed clones were verified by PCR amplification and Sanger sequencing using an ABI 3730xl DNA analyzer. Bacterial cultures were grown at 37°C in LB broth, supplemented with antibiotics as required: ampicillin (100 µg/mL), chloramphenicol (25 µg/mL), and kanamycin (50 µg/mL).

### Bacterial growth curve assays

Overnight cultures were diluted 1:1,000 into fresh LB broth or DMEM medium. A 200 μL aliquot of each diluted culture was dispensed into a 96-well microplate. The plate was incubated at 37°C with continuous shaking at 180 rpm for 24 h under aerobic conditions. Optical density at 600 nm (OD_600_) was recorded at 20 min intervals using an Agilent BioTek LogPhase 600 microplate reader (BioTek, USA). Experiments were performed with three biological replicates for each strain.

### Western blotting

Overnight cultures of WT and Δ*zraSR* strains were subcultured into fresh LB broth and grown to mid-log phase (OD_600_ = 0.6). The bacterial cells were harvested, washed twice with PBS (pH 7.4) by centrifugation at 5,000 rpm for 5 min, and resuspended in PBS. Cells were then lysed by sonication on ice, and the lysate (Beyotime; ST206) was centrifuged at 12,000 rpm for 15 min at 4°C. The supernatant was then mixed with SDS-PAGE loading buffer (Genstar; II181) to a final concentration of 1× and boiled at 95°C for 10 min. Proteins were separated by 12% SDS-PAGE and transferred to polyvinylidene difluoride membranes (Millipore; ISEQ00010). After blocking with 5% skim milk in TBST for 2 h at room temperature, the membranes were incubated overnight at 4°C with the following primary antibodies: anti-FLAG (1:1,000; Sigma; F1804) and anti-DnaK (1:1,000; Abcam; ab69617), or anti-CD48 (1:1,000; Abcam; ab134049) and anti-Hsp70 (1:1,000; Abcam; ab5439). Following three washes with TBST, membranes were incubated with an HRP-conjugated goat anti-mouse secondary antibody (1:5,000; Abcam; ab6789) for 1 h at room temperature. Protein bands were visualized using the Sparkjade ECL Plus detection system (Sparkjade Biotechnology; ED0016), and images were captured with an Amersham Imager 600 system (General Electric). Pre-stained protein molecular weight markers (GenStar; M228) were used. Signal intensities were quantified with ImageJ software and normalized to the DnaK loading control from three independent experiments.

### *Assay for* fimS *orientation*

The orientation of *fimS* was determined by PCR amplification combined with restriction enzyme digestion, following a previously published protocol [30]. In brief, genomic DNA extracted from stationary-phase cultures of WT and Δ*zraSR* strains bacterial genomic DNA extraction kit (Cwbio, CW0552S) following the manufacturer’s instructions. The extracted DNA was used as the template to amplify the invertible region, yielding a 601-bp PCR product. Equal amounts of the amplified DNA were digested with SnaBI (Thermo Fisher Scientific; FD0404), which produces distinct fragment patterns corresponding to the ON (403 bp and 198 bp) and OFF (440 bp and 161 bp) orientations. The digestion products were separated on a 2.5% agarose gel, stained with Gel-Red, and visualized under ultraviolet light.

### Cell adhesion and invasion assays

Cell adhesion and invasion assays were performed using the immortalized HBMEC line, kindly provided by Dr. K.S. Kim (Johns Hopkins University, Baltimore, MD). Cells were maintained in Dulbecco’s modified Eagle’s medium (DMEM; Gibco; 11,965,092) supplemented with 10% fetal bovine serum (FBS; Gibco; 10,100,147) and 10% Nu-Serum (Corning; 355,500) at 37°C in a 5% CO_2_ incubator.

For the adhesion assay, HBMECs were incubated with bacteria at a multiplicity of infection (MOI) of 100 for 1.5 h. Cells were then washed with sterile PBS and lysed using 0.5% Triton X-100 (Solarbio; T8200). Bacteria were collected and enumerated by plating onto LB agar plates.

For the invasion assay, following the 1.5 h incubation, cells were treated with DMEM containing 100 μg/mL gentamicin for 1 h to kill extracellular bacteria. Cells were then washed with sterile PBS and lysed using 0.5% Triton X-100. Bacteria were collected and enumerated by plating onto LB agar plates.

### Transwell assay

An *in vitro* endothelial barrier model was established using HBMECs cultured on collagen-coated Transwell inserts (3 µm pore polycarbonate membrane; Corning-Costar), following a previously described method with minor modifications [31]. Briefly, 2 × 10^5^ HBMECs were seeded into the upper chamber in 200 µL of DMEM supplemented with 10% FBS and Nu serum; the bottom chamber was filled with 600 µL of the same medium. The medium was changed every other day. Cells were grown to confluence over at least 7 days, and monolayer integrity was monitored by measuring trans endothelial electrical resistance (TEER) using an ECIS TEER24 system (Applied Biophysics Inc.).

For the bacterial translocation assay, log-phase bacteria were introduced into the cell layers on Transwell filters at MOI of 100 and incubated for 1.5 h. Both upper and bottom chambers were then washed with sterile PBS and incubated with DMEM containing 100 µg/mL gentamicin for 1 h to kill extracellular bacteria. After an additional PBS wash, both chambers were maintained in DMEM with 25 μg/mL trimethoprim (MedChemExpress; HY-B0510) and 50 μM rottlerin (MedChemExpress; HY-18980) for 4 h. Bacteria were collected and enumerated from the upper and bottom chambers by plating onto LB agar plates. Concurrently, cells on the Transwell filters were lysed with 0.5% Triton X-100, and bacteria were collected and enumerated by plating onto LB agar.

Bacterial transcytosis was calculated as the percentage of bacteria recovered from the lower chamber relative to the total number of bacteria recovered from all sections of the Transwell system (upper chamber, lower chamber, and cell lysate from the filter).

### RNA extraction and qRT-PCR

Total RNA was extracted using TRIzol reagent (Invitrogen, 15,596,026) following the manufacturer’s protocol and treated with RNase-free DNase I (Qiagen, 79,256) to remove genomic DNA. RNA concentration and quality were measured using a NanoDrop spectrophotometer (Thermo Fisher Scientific, USA). cDNA was synthesized from the extracted RNA (1 μg) using the StarScript III RT MasterMix kit (GenStar, China). qRT-PCR was then performed using SYBR Green PCR Master Mix (GenStar; A304) on an ABI 7500 thermocycler (Applied Biosystems, USA). Relative gene expression levels were calculated using the 2^−ΔΔCt^ method and normalized to the expression of the housekeeping gene *rpoA* in each experiment.

### RNA-seq analysis

Overnight-cultured bacteria of *E. coli* K1 WT and Δ*zraSR* strains were inoculated into fresh LB medium at a 1:100 dilution and cultured till the OD_600_ value reached approximately 0.6. Total RNA was extracted from the collected bacteria as described above. RNA quality was assessed with an Agilent 2100 Bioanalyzer (Santa Clara, CA) and agarose gel electrophoresis (AGE). Illumina library preparation, sequencing, and data analysis were performed by Majorbio (Shanghai, China). The sequencing reads were mapped to the *E. coli* K1 reference genome (CP007149.1). Gene expression levels were quantified and reported as FPKM. For differentially expressed genes (DEGs) analysis, raw read counts were normalized by TMM method. DEGs analysis was performed with the edgeR package, applying significance thresholds of |log2(fold change)| >1 and an adjusted *p*-values < 0.005 (Benjamini–Hochberg correction).

### Electrophoretic mobility shift assays (EMSAs)

The 6×His-tagged ZraR protein were expression and purified in *E. coli* BL21 (DE3). For competition assays, DNA fragments were amplified with or without 6-FAM-labeled primers and purified using the SPARKeasy Gel DNA Extraction Kit (Sparkjade; AE0101). The purified DNA probe (10 ng) was incubated with 1 μM 6×His-tagged ZraR protein at 37°C for 30 min in binding buffer (10 mM Tris-HCl pH 8.0, 50 mM KCl, 1 mM EDTA, 1 mM DTT, 50 μg/mL BSA, 10% glycerol) supplemented with 2 mM acetyl phosphate, and different concentrations of unlabeled DNA fragments (0 to 500 ng) were added. Protein-DNA complexes were separated on a 6% native polyacrylamide gel in 0.5×TBE buffer. Gels were visualized using an Amersham Imager 600 system (General Electric).

### DNase I footprinting assay

DNase I footprinting was performed as described previously [32]. The *fimS* region was amplified from *E. coli* K1 genomic DNA using a 6-FAM-labeled forward primer (5’ end modification) and an unlabeled reverse primer (primers listed in Supplementary Table S2). The 6-FAM-labeled DNA probe was incubated with purified 6×His-ZraR protein in binding buffer (10 mM Tris-HCl pH 8.0, 50 mM KCl, 1 mM EDTA, 1 mM DTT, 50 μg/mL BSA, 10% glycerol) for 30 min at 37°C. A control sample without ZraR was processed in parallel. The protein-DNA mixture was then partially digested with 0.05 units of DNase I (Sigma-Aldrich; AMPD1) for 10 min at 37°C. The reaction was stopped by heating at 85°C for 10 min. Samples were submitted to MAP Biotech Co., Ltd. (Shanghai, China) for fragment analysis on an ABI 3730xl DNA analyzer. Electropherograms were visualized and analyzed using Peak Scanner Software v2.0 (Applied Biosystems).

### Chromatin immunoprecipitation and quantitative PCR (ChIP-qPCR)

Bacteria were grown to mid-logarithmic phase, and protein expression was induced to with isopropyl-β-D-thiogalactoside (IPTG; Solarbio; II0130) at 37°C for 3 h. Bacteria were cross-linked with 1% formaldehyde for 25 min at room temperature and then reaction was quenched by adding 0.5 M glycine (Solarbio; IG0720). After centrifugation, bacterial pellets were three times washed with ice-cold PBS. The pellets were then resuspended in lysis buffer (50 mM Tris-HCl (pH 7.5), 1 mM EDTA, 100 mM NaCl, 1 mM PMSF, 20 mg/mL lysozyme) and incubated at 37°C for 30 min. Subsequently, an ultrasound buffer (100 mM Tris–HCl (pH 7.5), 200 mM NaCl, 1 mM EDTA, 2% v/v Triton X-100) was added, and the samples were sonicated to shear DNA into fragments ranging from 100 to 500 bp. After centrifugation at 12,000 rpm for 10 min, the supernatants were incubated overnight with anti-FLAG antibody (Sigma; F1804) followed by a 5-h incubation with protein A magnetic beads (Invitrogen; 10002D). The beads complexes were then incubated with elution buffer (50 mM Tris-HCl (pH 8.0), 10 mM EDTA, 1% w/v SDS) at 70°C for 10 min. The elution DNA samples were purified and performed qPCR using an Applied Biosystems ABI 7500. The *rpoS* gene (nonspecific enrichment) was used as a reference. A control sample processed without antibody (Mock-ChIP) served as the negative control. The relative enrichment was calculated using the 2^−ΔΔCt^ method and normalized to the housekeeping gene *rpoA* in each experiment.

### Animal experiments

2- to 5-day-old Sprague-Dawley rat pups were purchased from Beijing Vital River Laboratory Animal Technology Co., Ltd. (Beijing, China). The animal model used for colonization and survival analysis has been described in detail previously [33]. The pups were housed under specific pathogen-free (SPF) conditions at 22 ± 2°C with 50 ± 10% relative humidity and a 12-h light/dark cycle. Standard rodent chow and water were provided ad libitum throughout the experimental periods. Pups were randomly assigned to experimental groups using a random number table to minimize bias. For colonization experiments, pups were subcutaneously injected with 1 × 10^5^ CFU of the logarithmic bacteria. After 18 h post-infection (hpi), the rat pups were directly euthanized by decapitation, and blood and cerebrospinal fluid (CSF) samples were collected. Bacterial loads in these samples were quantified by culturing serial dilutions on LB agar plates. For survival analysis, a separate group of pups was similarly infected via subcutaneous injection with 1 × 10^5^ CFU of logarithmic bacteria, and their survival was monitored and recorded every 8 h for a total observation period of 96 h. All animal experiments were conducted according to ARRIVE guidelines.

### Hemagglutination (HA) assay

Bacterial strains were cultured statically in LB broth at 37°C for 24 h. Cells were normalized to an OD_600_ of 1.0, and 1 mL of culture was harvested by centrifugation at 5,500 rpm for 5 min. The pellet was resuspended in 100 μL of PBS. In a 96‑well round‑bottom microtiter plate, 25 μL of PBS was added to each well. Then, 25 μL of the bacterial suspension was added to the first well and mixed thoroughly. A two‑fold serial dilution was performed by transferring 25 μL from the first well to the second well (containing 25 μL PBS), mixing, and continuing the series. Finally, 25 μL of a 1% (v/v) guinea pig erythrocyte suspension in PBS was added to each well. The plate was gently mixed and incubated at 4°C for 12 h. The HA titer was determined as the reciprocal of the highest dilution showing visible erythrocyte agglutination. Each assay was performed in triplicate.

### Statistical analysis

Data are presented as bar graphs or dot plots, displaying the mean ± standard deviation (SD) from three independent experiments. All statistical analyses were performed using GraphPad Prism 9.6.1 software. Based on the experimental design and data distribution as detailed in the respective figure legends, comparisons were made using the appropriate tests, including the two-tailed unpaired Student’s *t*-test, one-way ANOVA, two-way ANOVA, log-rank (Mantel-Cox) test, or the Mann–Whitney *U* test. Differences were considered significant at *p* < 0.05.

## Results

### *ZraSR TCS contributes to* E. coli *K1 penetration of the blood−brain barrier*

The ZraSR consists a TCS in *E. coli*, in which ZraS is the histidine sensor kinase and ZraR is the response regulator. By analyzing the publicly available RNA-seq data from *E. coli* K1 during infection of HBMECs, we found that the regulator ZraR was significantly upregulated [27], indicating that ZraSR may contribute the *E. coli* K1 penetration of the BBB.

**Figure 1.**
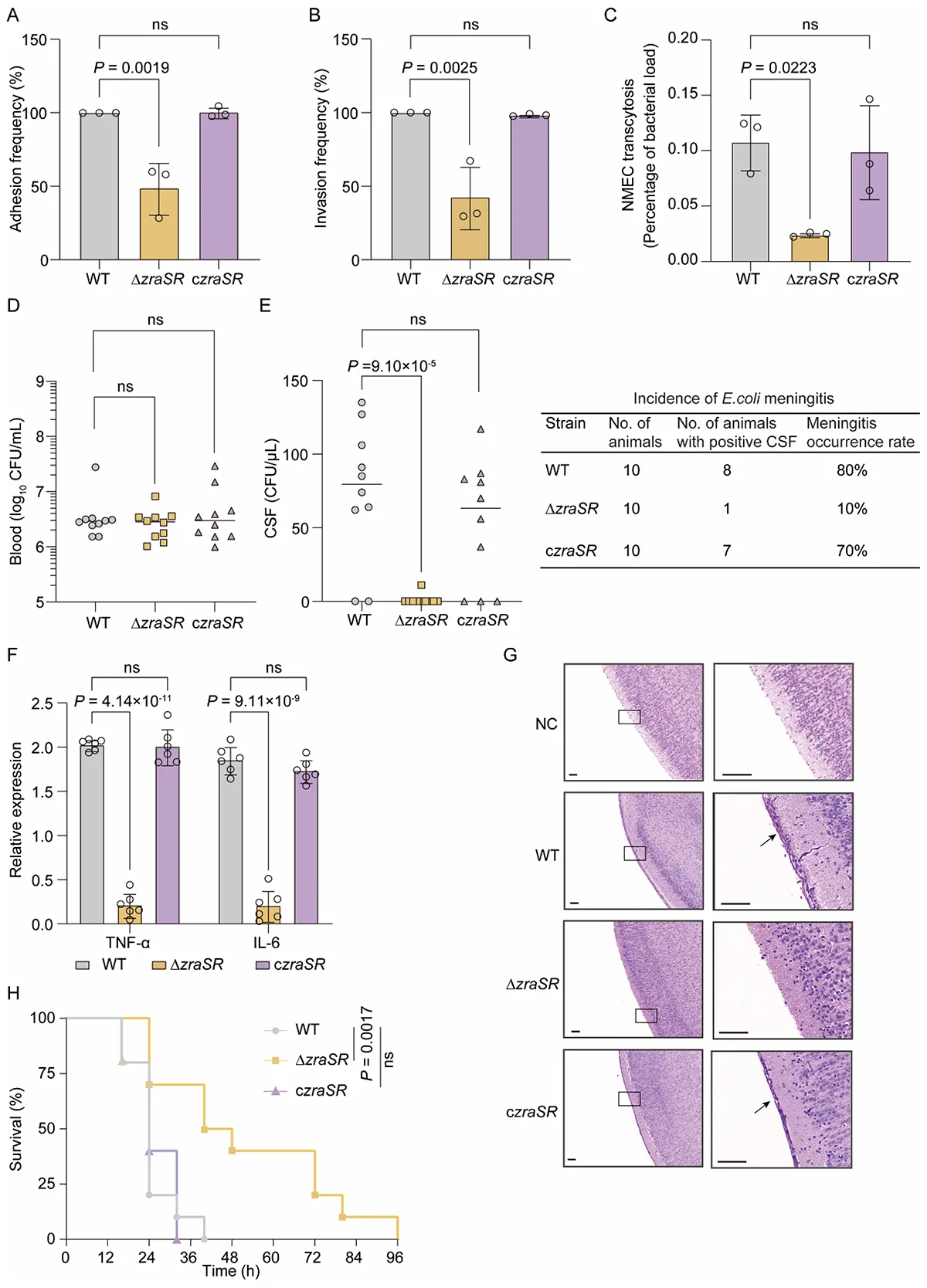
*zraSR* promotes the virulence of *E. coli* K1. (A-B) bacterial adhesion (A) or invasion (B) of HBMECs infected with WT, *∆zrasr* or *∆zrasr* complemented (c*zraSR*) strains. *n* = 3 independent experiments. (C) transcytosis of HBMECs with WT, *∆zrasr* or c*zraSR* strains. *n* = 3 independent experiments. (D-E) bacterial burdens in the blood (CFU/mL, D) and cerebrospinal fluid (CSF, CFU/μL) (E) were determined by 2- to 5-day-old rats subcutaneously received with 1 × 10^5^ CFU of the WT, Δ*zraSR*, or c*zraSR* strain at 18 hpi. CSF culture positivity was defined as meningitis. *n* = 10 rats per group. (F) the levels of TNF-α and IL-6 in the CSF of 2- to 5-day-old rats subcutaneously infected with 1 × 10^5^ CFU of WT, Δ*zraSR*, or c*zraSR* strain at 18 hpi. *n* = 6 rats per group. (G) H&E staining of brain sections was performed using uninfected healthy (negative control, NC) 2- to 5-day-old rats and littermates subcutaneously infected with 1 × 10^5^ CFU of WT, Δ*zraSR*, or c*zraSR* strain at 18 hpi. The right panels are higher-magnification images of the boxed regions, showing meningeal thickening and neutrophil infiltration (arrows). *n* = 3 rats per group. Scale bar, 100 μm. (H) survival curves of 2- to 5-day-old rats subcutaneously injected with 1 × 10^5^ CFU of the WT, Δ*zraSR* or c*zraSR* strain. *n* = 10 rats per group. ns, no significance. In (A-C and F), the data are presented as mean ± SD. One-way ANOVA (A-C), Mann-Whitney *U* test (D-F), the log-rank (mantel-cox) test (H) were applied.

To determine whether ZraSR contributes to *E. coli* K1 virulence, we constructed a *zraSR* mutant strain (Δ*zraSR*) using the λ-Red recombination system. Growth curves analysis showed that WT and Δ*zraSR* exhibited similar growth rates when they were grown in LB broth and DMEM media (Figs. S1A-B). We next examined whether Δ*zraSR* is defective for adhesion to and invasion of HBMECs. The results showed that the adhesion and invasion frequencies were significantly decreased in Δ*zraSR* compared to the WT strain (Figure 1(A,B)). The *zraSR* complementation by plasmid which constructed the *∆zraSR*-complemented strain (c*zraSR*) restored the adhesion and invasion frequencies to WT levels (Figure 1(A,B)). Furthermore, using a polarized HBMEC monolayer with tight junctions in Transwells, which mimics the BBB *in vitro* [34], we found that deletion of *zraSR* significantly decreased the transcytosis of *E. coli* K1 (Figure 1(C)). Therefore, ZraSR TCS contributes to *E. coli* K1 penetration of the BBB.

To determine the potential role of *zraSR* in the development of bacteremia and meningitis *in vivo*, the neonatal rats were subcutaneously inoculated with WT, Δ*zraSR* and *∆zraSR*-complemented strains. Blood and CSF samples were collected at 18 h post-infection and cultured on the LB agar plate to assess bacteremia and meningitis, respectively. The results revealed that the bacterial burdens in the blood of rats infected with Δ*zraSR* strain showed no significant difference compared with those infected with WT or *∆zraSR*-complemented strains (Figure 1(D)). In contrast, the incidence of meningitis – defined as positive CSF culture – in Δ*zraSR*-infected rats was 10%. By comparison, the incidence of meningitis in rats infected with WT and *∆zraSR*-complemented strain showed 80% and 70%, respectively, both of which were higher than those infected with Δ*zraSR* (Figure 1(E)). Consistently, the bacterial burdens in the CSF of rats infected with Δ*zraSR* strain showed significantly lower than those infected with WT or ∆*zraSR*-complemented strains (Figure 1(E)). These results suggested that the *zraSR* contributes to *E. coli* K1 penetration of the BBB and development of meningitis within the host. Moreover, we also detected the levels of IL-6 and TNF-α in CSF which was collected from rats infected with WT, Δ*zraSR* and *∆zraSR*-complemented strains. We found that the levels of both TNF-α and IL-6 were significantly lower in CSF of Δ*zraSR*-infected rats compared with those infected with either the WT or *∆zraSR*-complemented strain (TNF-α: WT vs Δ*zraSR*, *p* = 4.14 × 10^−11^; IL-6: WT vs Δ*zraSR*, *p* = 9.11 × 10^−9^) (Figure 1(F)). Furthermore, hematoxylin and eosin (H&E) staining showed that uninfected healthy control rats (Negative Control, NC) exhibited normal meningeal structure with no inflammatory cell infiltration. In contrast, brain tissues from rats infected with WT and *∆zraSR*-complemented strains revealed thickened meninges accompanied by neutrophil infiltration, whereas no discernible histopathological changes were observed in the meninges of the rats infected with Δ*zraSR* (Figure 1(G)). Survival curves showed that the neonatal rats infected with Δ*zraSR* exhibited prolonged survival times compared to those infected with WT and *∆zraSR*-complemented strains (Figure 1(H)). Collectively, these results indicated that *zraSR* contributes to the pathogenicity of *E. coli* K1 by facilitating its penetration of the BBB, a critical step for development of meningitis.

### ZraSR TCS positively regulates the type 1 fimbriae expression

**Figure 2.**
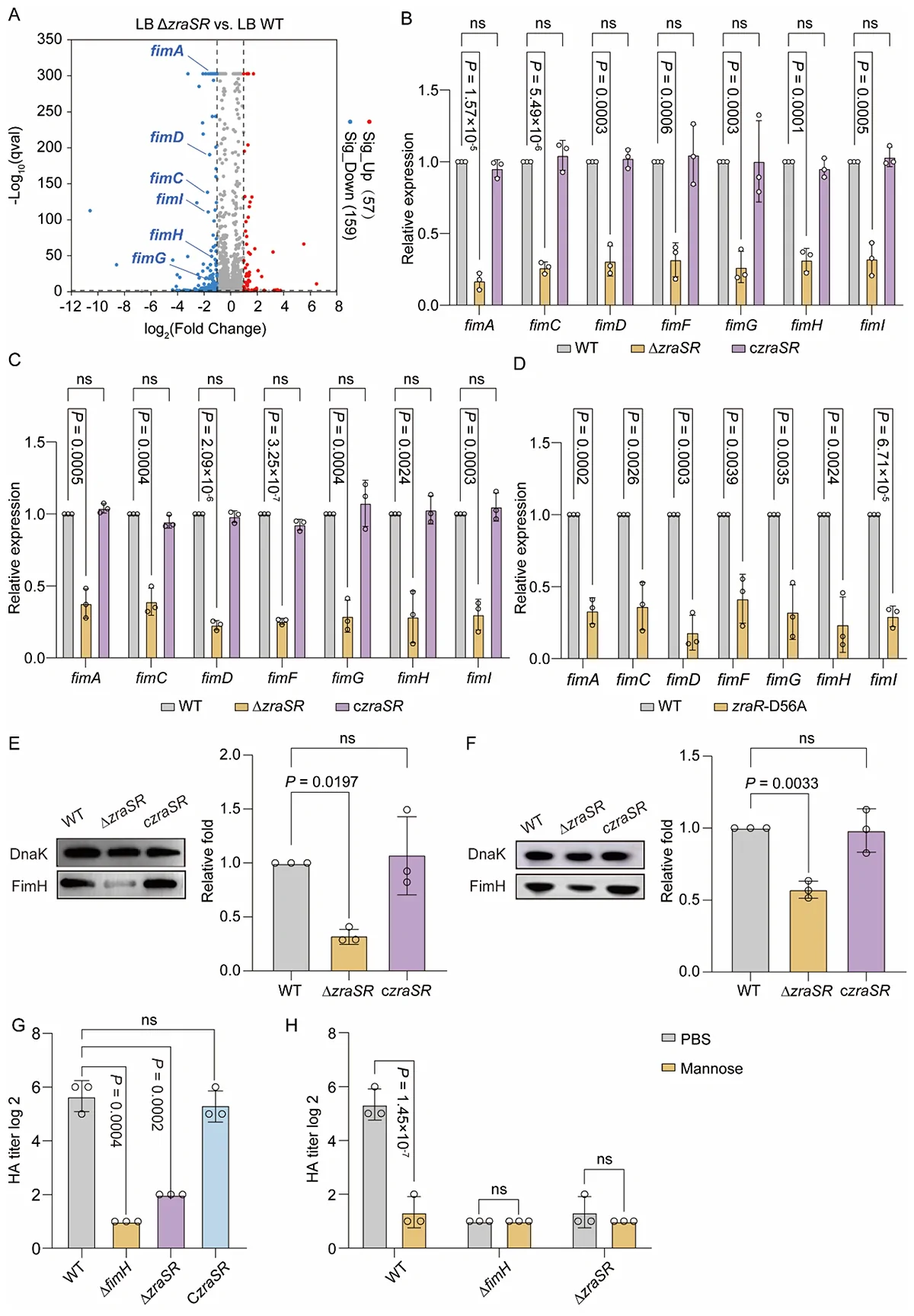
ZraSR TCS positively regulates type 1 fimbriae expression. (A) Volcanoplot showing the differentially expressed genes in Δ*zraSR* vs WT grown in LB broth. (B) qRT-PCR analysis of *fim* genes expression in WT, Δ*zraSR* and c*zraSR* strains in LB broth. *n* = 3 independent experiments. (C) qRT-PCR analysis of *fim* genes expression in WT, Δ*zraSR* and c*zraSR* strains during infection of HBMECs. *n* = 3 independent experiments. (D) qRT-PCR analysis of *fim* genes expression in WT and *zraR*-D56A strains in LB broth. *n* = 3 independent experiments. (E-F) Western blot analysis of FimH-3×FLAG expression in WT, Δ*zraSR*, or c*zraSR* strains grown in LB broth (E) or during infection with HBMECs (F). *n* = 3 independent experiments. DnaK was used as the loading control. (G) HA assays of the production of type l fimbria in WT, Δ*fimH*, Δ*zraSR* and c*zraSR* strains. *n* = 3 independent experiments. (H) HA assays of the production of type l fimbria in WT, Δ*fimH* and Δ*zraSR* strains in the presence or absence of 3% mannose. *n* = 3 independent experiments. ns, no significance. In (B-F), the data are presented as mean ± SD. Two-tailed Student’s *t* test (B-D), one-way ANOVA (E-G), two-way ANOVA (H) were applied.

To investigate the molecular mechanism by which *zraSR* regulates the virulence of *E. coli* K1, transcriptome analysis was performed on WT and Δ*zraSR* strains. The transcriptome results showed that 57 genes and 159 genes were up-regulated and down-regulated in Δ*zraSR*, respectively, compared with the WT strain (Figure 2(A) and Supplementary Data 1). Furthermore, we found that most genes that encoded type 1 fimbriae (*fimAICDFGH*) were significantly down-regulated in Δ*zraSR* compared with WT (Figure 2(A)). Therefore, we performed qRT-PCR assay to determine the expression of type 1 fimbriae in WT and *∆zraSR* strains. The result showed that all seven genes encoding type 1 fimbriae were significantly decreased in Δ*zraSR* compared with WT grown in LB broth (Figure 2(B)). As specificity controls, *glpK* and *secY*, whose expression was unaffected by *zraSR* deletion in our RNA-seq data (Supplementary Data 1), were confirmed by qRT-PCR to be unchanged in the Δ*zraSR* strain compared with the WT strain (Figure S2A), suggesting that RNA-seq data were reliable in identifying transcriptional changes. In addition, we also detected the expression of type 1 fimbriae in WT and Δ*zraSR* during infection. Consistent with the result of strains grown in LB broth, all seven genes encoding type 1 fimbriae were significantly decreased in Δ*zraSR* compared with WT during infection of HBMECs, suggesting the positive regulation of type 1 fimbriae genes by ZraSR (Figure 2(C)).

Upon recognition of specific external stimuli, the response regulator of a TCS is activated by phosphorylation and undergoes a conformational change and frequently homodimerizes, which enables it to bind to target promoters and subsequent regulation of downstream gene expression [35]. Therefore, we constructed a point mutant strain that eliminated the phosphorylation site of ZraR with asparticacid-to-alanine (*zraR*-D56A). We found that compared with WT strain, the expression of type 1 fimbriae was significantly decreased in *zraR*-D56A strain, suggesting that the phosphorylation is required for ZraR activation and the expression of type 1 fimbriae (Figure 2(D)).

*E. coli* K1 binding to HBMECs exploits type 1 fimbriae in particular FimH to interact with CD48 on HBMEC. To determine the expression of FimH at the protein level, a 3×FLAG C-terminal fusion construct of *fimH* was generated. Western blotting revealed that the expression of FimH was significantly down-regulated in Δ*zraSR* compared with WT both in the LB broth and during infection with HBMECs (Figure 2(E,F)). Accordingly, *zraR*-D56A strain also exhibited markedly reduced FimH protein levels in LB broth and during HBMECs infection compared with those infected with WT, respectively (Figs. S2B-C). These results confirmed that ZraSR promotes the expression of type 1 fimbriae in *E. coli* K1.

In addition, hemagglutination (HA) assays were performed to verify the effect of ZraSR on the surface production of functional type 1 fimbriae. The results showed a significant decrease in HA titer in Δ*zraSR* compared with WT strain, and *∆zraSR*-complemented strain restored the HA phenotype to WT levels (Figure 2(G)). The Δ*fimH* strain, which lacks functional type 1 fimbriae, showed barely detectable hemagglutination [36]. Furthermore, the difference in HA titer between WT and Δ*zraSR* was abolished in the presence of mannose, a competitive inhibitor of type 1 fimbriae-mediated adhesion (Figure 2(H)). These results confirmed that ZraSR is required for the surface assembly and production of functional type 1 fimbriae in *E. coli* K1.

### *ZraSR promotes* E. coli *K1 invasion via* fimH

**Figure 3.**
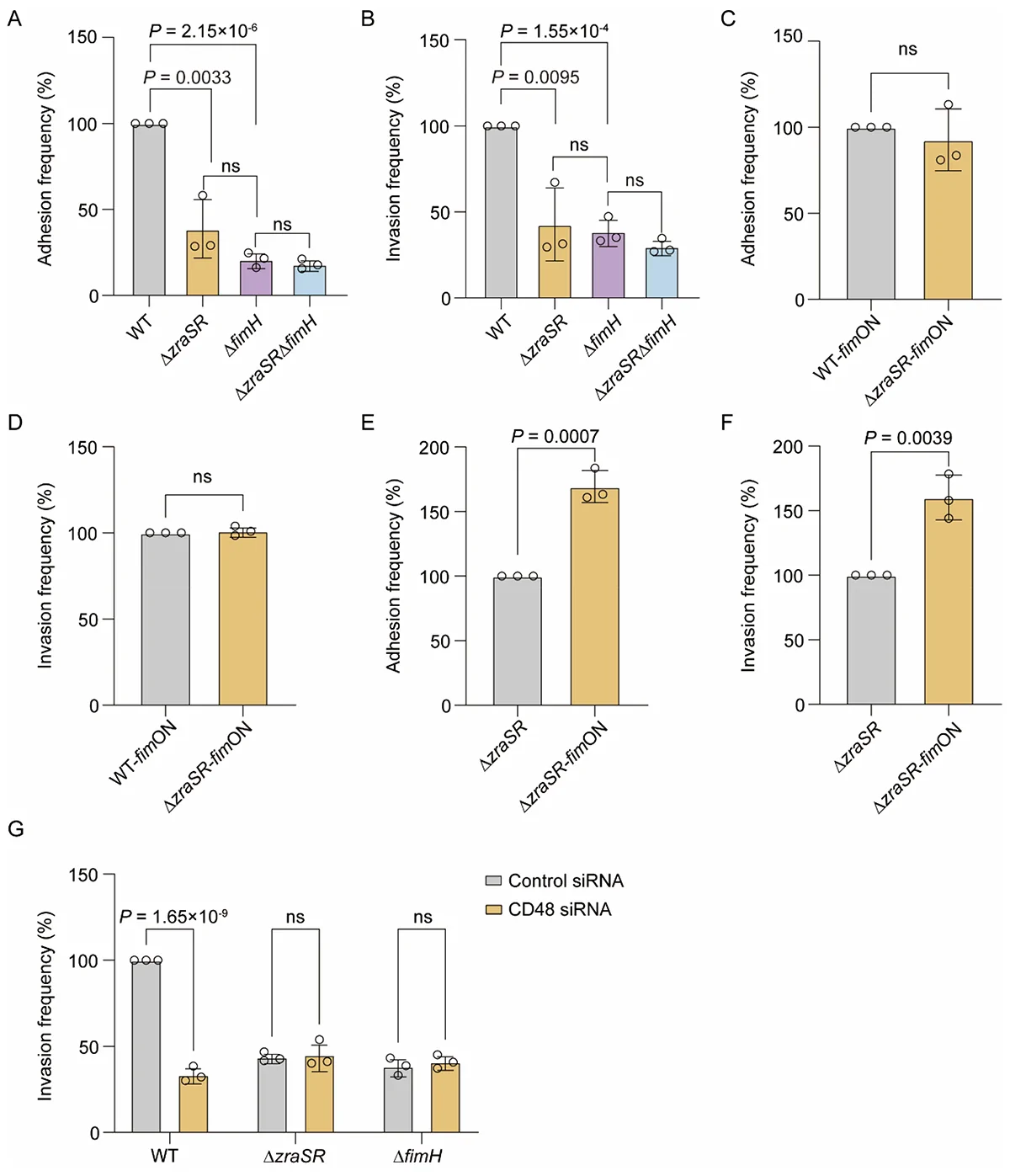
ZraSR promotes *E. coli* K1 invasion via *fimH*. (A-B) bacterial adhesion (A) and invasion (B) of HBMECs infected with WT, ∆*zraSR, ∆fimh* or ∆*zraSR*∆*fimH* strains. *n* = 3 independent experiments. (C-D) bacterial adhesion (C) and invasion (D) of HBMECs infected with WT-*fim*ON or ∆*zraSR-fim*ON strains. *n* = 3 independent experiments. (E-F) bacterial adhesion (E) and invasion (F) of HBMECs infected with ∆*zraSR* or ∆*zraSR-fim*ON strains. *n* = 3 independent experiments. (G) bacterial invasion of control and CD48-knockdown HBMECs infected with WT, *∆zrasr* or *∆fimh* strains. *n* = 3 independent experiments. ns, no significance. In (A-G), the data are presented as mean ± SD. One-way ANOVA (A-B), two-tailed Student’s *t*-test (C-F), two-way ANOVA (G) were applied.

To confirm the role of *fimH* in mediating *E. coli* K1 adhesion to and invasion of HBMECs, we constructed a Δ*fimH* deletion mutant and a double-knockout strain Δ*fimH*Δ*zraSR*. We simultaneously performed adhesion and invasion assays using WT, Δ*zraSR*, Δ*fimH* and Δ*fimH*Δ*zraSR* strains. Compared with the WT, the results showed that both Δ*zraSR* and Δ*fimH* was defective for adhesion to and invasion of HBMECs which consistent with the published results [14] (Figure 3(A,B)). Moreover, the adhesion to and invasion frequency of Δ*zraSR* and Δ*fimH* were no significant difference (Figure 3(A,B)). More importantly, the double mutant Δ*fimH*Δ*zraSR* did not display further aggravated adhesion and invasion defects (Figure 3(A,B)), suggesting that *zraSR* deletion did not affect its function in a Δ*fimH* background. In addition, we also constructed the *fimS* phase-locked-ON strains in WT and Δ*zraSR* background, respectively, in which the inversion is blocked allowing the *fimS* promoter element to be persistently reoriented into the ON orientation [37]. We found that there is no significant difference between WT-*fim*ON and Δ*zraSR-fim*ON in the adhesion and invasion frequencies of HBMECs (Figure 3(C,D)), suggesting that *zraSR* deletion did not confer additional invasion defects in the *fimS* phase-locked-on background. Importantly, we further compared the adhesion and invasion frequencies of the Δ*zraSR-fim*ON strain with those of the Δ*zraSR* strain (without phase locking). The results showed that locking *fimS* in the ON position significantly restored both adhesion and invasion capabilities in the Δ*zraSR* background (Figure 3(E,F)). Collectively, these data provide direct genetic evidence that the loss of ZraSR function can be fully compensated by locking the *fim* operon in the ON orientation, further supporting that ZraSR promotes *E. coli* K1 invasion via type 1 fimbriae. Previous studies have shown that FimH interacts with CD48 present on the HBMEC surface which involved in the binding to and invasion of HBMECs in *E. coli* K1 [10]. To further examine the invasion of HBMECs mediated by ZraSR-FimH regulation pathway, endogenous CD48 was knocked down using specific siRNAs, as confirmed by Western blot showing an approximately two-fold decrease in CD48 protein expression (Fig. S3). This knockdown led to a significant decrease (2.98-fold) of invasion rate in WT strain compared with that achieved siScr control (Figure 3(G)). In contrast, the already low invasion of the Δ*fimH* was not further reduced by CD48 depletion (Figure 3(G)), formally demonstrating that CD48 promotes bacterial internalization specifically through FimH. Similarly, CD48 knockdown did not affect invasion in the Δ*zraSR*, and the invasion levels of Δ*zraSR* remained indistinguishable from those of Δ*fimH* under both siScr and siCD48 conditions (Figure 3(G)). These results indicated that ZraSR promotes *E. coli* K1 invasion of HBMECs via FimH-CD48 pathway, and that loss of either ZraSR or FimH renders the bacteria insensitive to CD48 perturbation.

### *ZraR interacts with* fimS *directly resulting in the bias toward the ON orientation*

**Figure 4.**
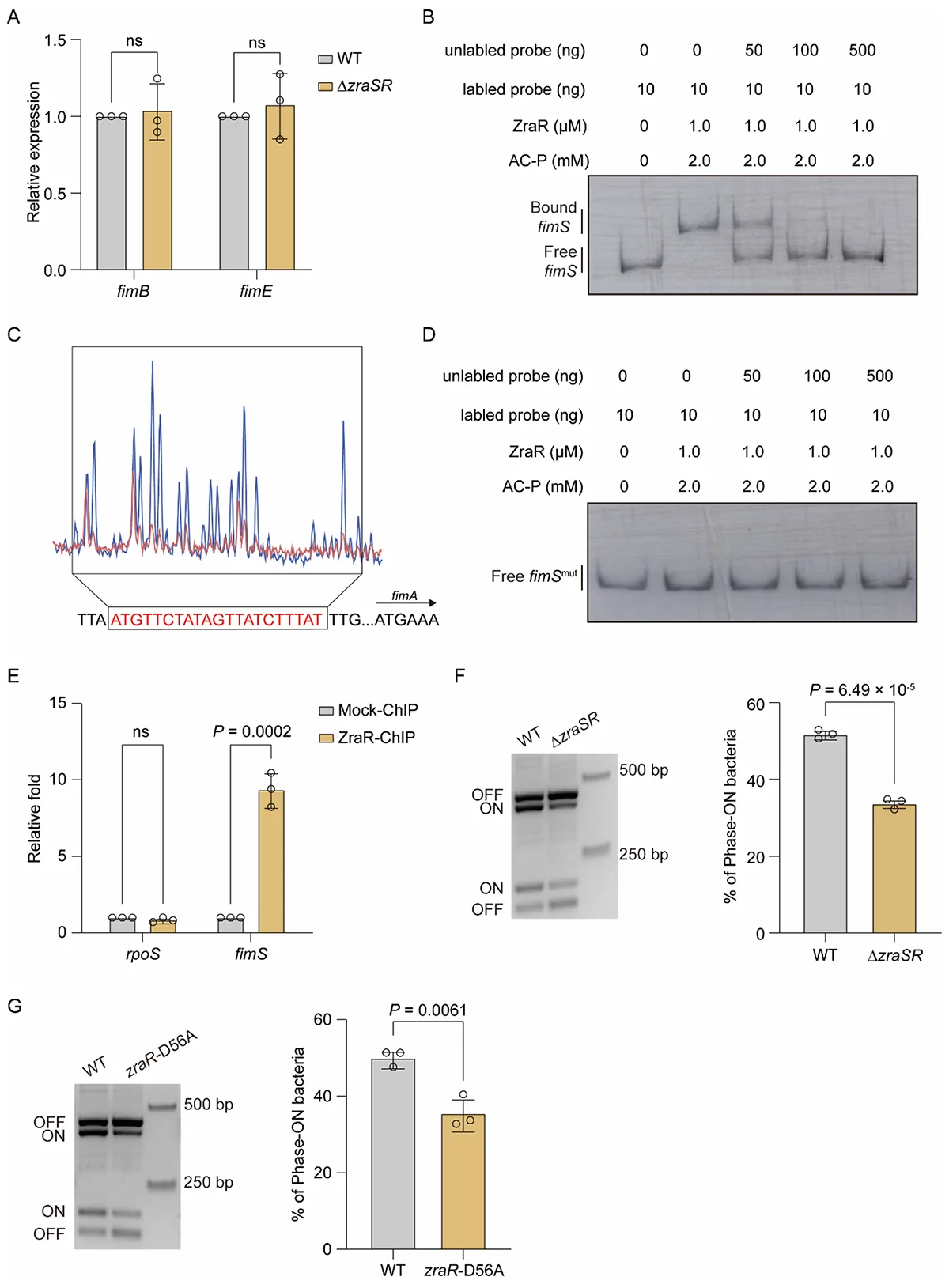
ZraR interacts with *fimS* directly resulting in the bias toward the ON orientation. (A) qRT-PCR analysis of the *fimB* and *fimE* expression in WT and ∆*zraSR* strain in LB broth. *n* = 3 independent experiments. (B) Competitive EMSA of the *fimS* DNA fragment with purified ZraR protein. (C) DNase l footprinting assay identified precise ZraR binding sites on *fimS*. The protected region shows a significant reduced peak intensity (red) pattern compared with that of the control (blue). (D) Competitive EMSA of the mutant *fimS* DNA fragment with purified ZraR protein. (E) the Fold enrichment for *fimS* promoter and the negative control (*rpoS*) in ZraR-ChIP assays. *n* = 3 independent experiments. (F-G) Quantification of the percentage of bacteria with *fimS* in the phase-ON orientation of WT and ∆*zraSR* (F) or WT and *zraR*-D56A (G). *n* = 3 independent experiments. ns, no significance. In (A and E-G), the data are presented as mean ± SD. Two-tailed Student’s *t*‑test (A and E-G) were applied.

To determine whether ZraSR induced the expression of type 1 fimbriae via *fimS* directly or via *fimB* and *fimE* that specifically catalyze the site-specific DNA inversion responsible for phase variation, we rechecked the transcriptome results and found that the expression of *fimB* and *fimE* showed no significant difference between WT and Δ*zraSR*. qRT-PCR confirmed that deletion of ZraSR did not affect the expression of *fimB* and *fimE* (Figure 4(A)). Subsequently, we investigated whether ZraSR activates type 1 fimbriae by directly binding to *fimS*. Competitive EMSAs showed that with increasing concentrations of ZraR, slowly migrating bands were observed for the FAM-labled *fimS* DNA. The addition of unlabeled *fimS* DNA effectively competed for ZraR binding with the labeled DNA, resulting in the loss of the shifted complex (Figure 4(B)). However, no migrating band was observed for the DNA fragment derived from the FAM-labled *rpoS* gene which served as the negative control (Fig. S4). To identify the precise ZraR-binding motif on *fimS*, we performed DNase I footprinting assay. The result showed that ZraR protects a 21-base pair motif (5’-ATGTTCTATAGTTATCTTTAT-3’) in the *fimS* phase-ON orientation (Figure 4(C)). Mutation (5’-ATAAAGATAACTATAGAACAT-3’) of this identified binding site completely abolished the binding of ZraR to *fimS* (Figure 4(D)), indicating ZraR binds to *fimS in vitro* specifically. In agreement with the EMSA results, we performed ChIP-qPCR and found that the *fimS* showed a 9.36-fold enrichment in ZraR-ChIP samples compared with mock-ChIP control samples (Figure 4(E)). Collectively, these results indicated that ZraR binds to *fimS* both *in vitro* and *in vivo*.

To determine whether *zraR* promoted the phase-ON orientation of the type 1 fimbriae, we performed phase switch orientation assay which investigates the orientation of the phase switch by PCR amplification and SnaBI restriction [36]. Due to the asymmetric positioning of the SnaBI cleavage site within the invertible element, the resulting fragment sizes and band intensities allowed us to estimate the proportion of bacteria in the phase-OFF state, reflecting transcriptional activity of the type 1 fimbrial operon [30,36]. We found that compared with WT strain, only 52.67% of *fimS* was in the phase-ON orientation in Δ*zraSR*, significantly lower than that in WT strain (Figure 4(F)). Consistently, we found that the proportion of *fimS* switched to the phase-ON orientation in *zraR*-D56A strain was significantly lower than that in WT strain (Figure 4(G)). These results indicated that the TCS ZraSR, upon phosphorylation, facilitates *fimS* inversion to the phase-ON orientation.

### ZraSR is activated by a mechanical cue generated by initial host adherence

**Figure 5.**
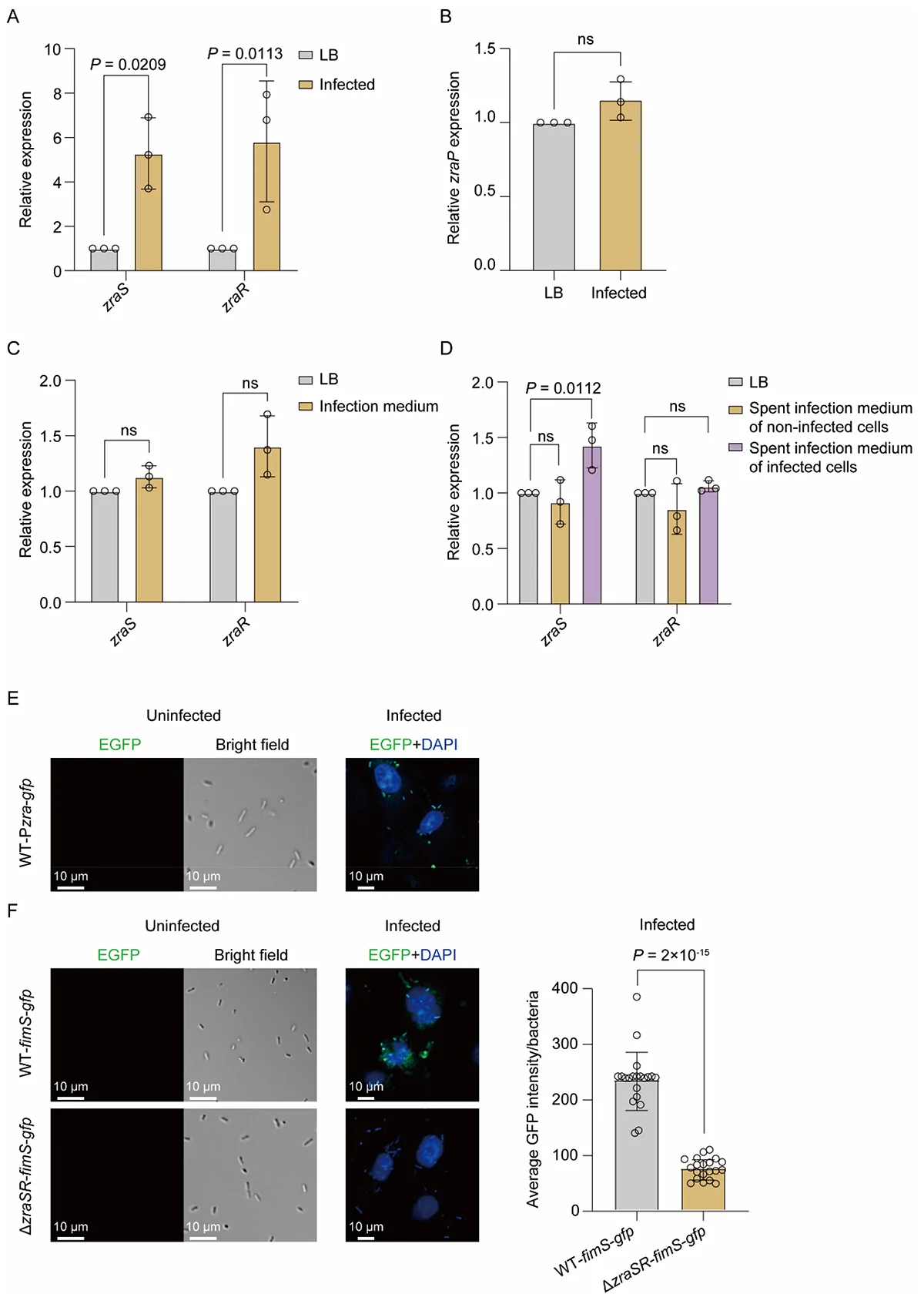
ZraSR is activated by a mechanical cue generated by initial host adherence. (A-B) qRT-PCR analysis of the *zraSR* (A) or *zraP* (B) expression in *E. coli* K1 grown in LB broth and during HBMECs infection. *n* = 3 independent experiments. (C) qRT-PCR analysis of the *zraSR* expression in *E. coli* K1 grown in LB broth and infection medium. *n* = 3 independent experiments. (D) qRT-PCR analysis of the *zraSR* expression in *E. coli* K1 grown in LB broth, infection medium of non-infected HBMECs and infection medium of infected HBMECs. *n* = 3 independent experiments. (E) representative images of WT-P*zra-gfp* reporter strains in uninfected vs infected HBMECs. Scale bar, 10 μm. (F) representative images and quantification of the average GFP intensity per bacterium of WT-*fimS-gfp* and Δ*zraSR-fimS-gfp* reporter strains in uninfected vs infected HBMECs. DNA was stained with DAPI and reporter activation was detected by GFP. Scale bar, 10 μm. ns, no significance. In (A-D and F) the data are presented as mean ± SD. Two-tailed Student’s *t* test (A-D and F) were applied.

To identify the mechanism of *zraSR* expression regulation, we first detected the expression of *zraS* and *zraR* during infection. We found that the expression of *zraS* and *zraR* was significantly upregulated compared with that grown in LB broth (Figure 5(A)). Moreover, we also detected the expression of *zraP*, which acts as a repressor of ZraSR [38]. We found that the expression of *zraP* showed no significant difference during infection compared with that grown in LB broth, suggesting that the upregulation of *zraSR* was not related to the expression of *zraP* (Figure 5(B)). Previous studies have demonstrated that the ZraSR is activated through Zn^2+^ binding to the periplasmic domains of ZraS. This metal ion binding induces a conformational change in the sensor histidine kinase, enabling the transfer of a phosphoryl group from the histidine kinase domain to the response regulator ZraR [39]. The optimal concentration of Zn^2+^ to induce ZraSR expression is 500 μM, which is much higher than the level of Zn^2+^ in cell cultures of infection medium (Ham’s F12: Medium 199–1× Earle salts [1:1], 5% heat-inactivated fetal bovine serum, 1% Na pyruvate, 0.5% glutamine) [38]. To determine whether the level of Zn^2+^ in infection medium activates the expression of *zraS* and *zraR*, we performed the qRT-PCR to detect the expression of *zraS* and *zraR* in infection medium compared to LB broth. We found that *zraS* and *zraR* expression showed no significant difference between these two conditions (Figure 5(C)), suggesting the activation of *zraS* and *zraR* is not due to the level of Zn^2+^ in infection medium. Then we detected the expression of *zraS* and *zraR* in spent infection medium of infected and non-infected HBMEC, and found the expression of *zraS* and *zraR* showed no significant difference in spent infection medium of infected or non-infected HBMEC compared with that grown in LB broth (Figure 5(D)). This result suggested that host produced substrates released into the infection medium have no effects on the expression of *zraSR*. As specificity controls, the transcriptional levels of *ntpA* and *gcvR* remained stable and showed no obvious alterations among all detected groups, including bacteria cultured in LB medium, infection medium and during HBMEC infection (Figs. S5A-C). These results confirmed that the increased expression of *zraR* and *fim* cluster genes upon host cell contact is a specific biological response triggered by bacterial adherence to host cells, rather than a nonspecific transcriptional alteration induced by culture medium components or host-derived soluble factors.

ZraSR involved in ESR, coordinately with sigma E, phage shock protein (Psp), CpxAR, and BaeSR, dedicated to the detection and repair of damaged components [25]. Previous study demonstrated that BaeSR was activated upon initial host cell adherence and induced the expression of locus of enterocyte effacement (LEE) genes in EHEC, thereby enhancing its virulence [28]. To identify whether a mechanical cue generated by initial host adherence induces the expression of ZraSR, we generated the GFP reporter strains of WT by introducing a P_*zra*_-*gfp* transcriptional fusion. HBMECs were infected with WT-P_*zra*_-*gfp* reporter strain, and *zra* promoter induction was analyzed in situ using fluorescence microscopy. We found that the significant GFP fluorescence was detected in the HBMEC-attached WT-P_*zra*_-*gfp* reporter strain, whereas nearly no GFP fluorescence was observed in non-attached bacteria (Figure 5(E)). Additionally, we found that the significant GFP fluorescence was detected in the HBMEC-attached WT-*fimS-gfp* reporter strain compared with the Δ*zraSR-fimS-gfp* strain, whereas nearly no GFP fluorescence was observed in non-attached bacteria in WT-*fimS-gfp* or Δ*zraSR-fimS-gfp* strain (Figure 5(F)). Moreover, the cell prior to treatment with PFA also showed significant GFP fluorescence in the HBMEC-attached WT-*fimS-gfp* reporter strain compared with the Δ*zraSR-fimS-gfp* strain, whereas nearly no GFP fluorescence was observed in non-attached bacteria in WT-*fimS-gfp* or Δ*zraSR-fimS-gfp* strain (Fig. S5D). These results suggested that the direct contact with host cells is the signal for activated the expression of ZraSR and type 1 fimbriae, facilitating the invasion and penetration of *E. coli* K1 to HBMECs.

## Discussion

**Figure 6.**
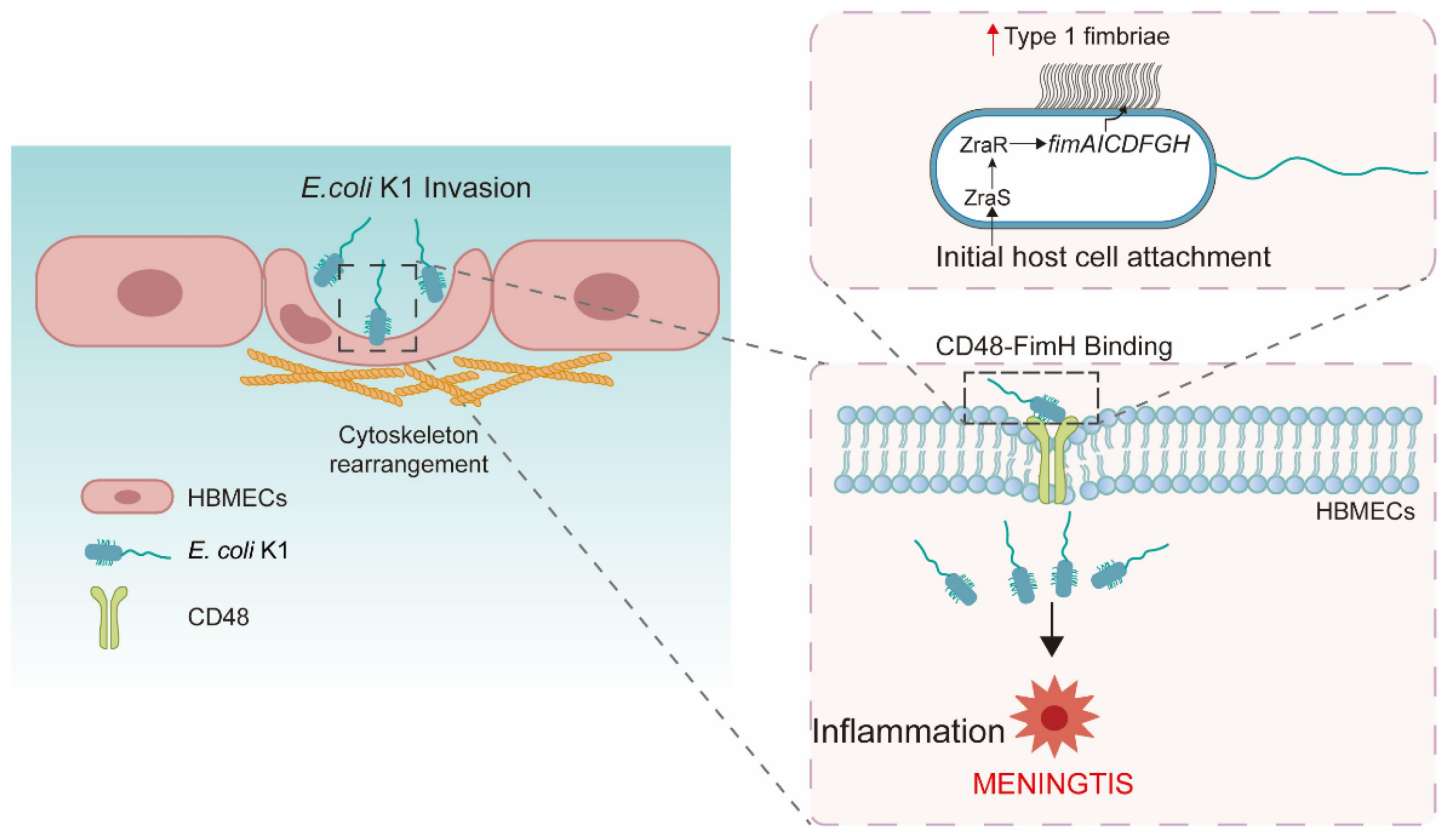
Model of ZraSR‑dependent type 1 fimbriae activation in *Escherichia coli* K1 meningitis. We proposed that upon attachment to HBMECs, a mechanical cue activates the ZraSR two-component system. Phosphorylated ZraR directly binds to *fimS*, driving phase-ON switching and type 1 fimbriae (FimH) expression. FimH interaction with host CD48 triggers cytoskeleton rearrangement and inflammation, enabling *E. coli* K1 to invade HBMECs and cause meningitis.

To survive in dynamic and hostile environments characterized by rapid fluctuations, bacteria must employ sophisticated adaptive strategies. These include the deterministic signal-responsive TCSs [40] and the stochastic, bet-hedging mechanism of phase variation [41,42]. Here, we report a unique integration of these two strategies in *E. coli* K1. We elucidate a complete pathway where the ZraSR TCS responses to a mechanical cue – host cell attachment – into the direct regulation of type 1 fimbriae phase variation. Specifically, mechanical stimuli trigger the phosphorylation of ZraR, which subsequently binds directly to the invertible element *fimS*, promoting its switch to the phase-ON orientation. This ZraSR-mediated upregulation of *fimH* enhances bacterial adhesion to and invasion of HBMECs, thereby facilitating penetration of the BBB and the progression of meningitis (Figure 6).

In pathogenic bacteria, TCSs are precisely activated within the complex host milieu by a diverse array of signals [43]. For instance, the PmrBA and QseBC TCSs jointly respond to Fe^3+^, enabling the UPEC to coordinate a transcriptional response that promotes intrinsic tolerance to the antibiotic polymyxin B [44]. The TCSs of SsrAB and PmrBA in *Salmonella* can be disrupted by a small-molecule inhibitor-dephostatin, suppressing virulence and restoring susceptibility to colistin antibiotic [45]. These TCSs represent canonical ESR mechanisms, wherein specific TCSs sense discrete signals to coordinate bacterial adaptation. Our findings position the TCS ZraSR is also the one of ESR systems. While ZraSR can be activated by high concentrations of metal ions like Zn^2+^ *in vitro* [39,46], our gene expression analysis under host-mimicking conditions, consistent with previous work [25], ruled out Zn^2+^ as the physiologically relevant signal (Figure 5). Instead, we demonstrated that the ZraSR ESR is activated specifically upon host cell attachment [28,47]. Ionic strength, osmolarity, pH, nutrient availability, attachment of pili and flagella, and envelope stress have been shown to be relevant for surface sensing [48]. The mechanism exploited by ZraSR shares a conceptual similarity with the CpxAR [49] system, which is activated by adhesion to hydrophobic surfaces, and the BaeSR [50] pathway, which responds to envelope-damaging agents [28,51], underscoring the role of ESRs in sensing physical and chemical threats to the envelope. Notably, Cpx and Bae respond to the fluctuation of the outer-membrane proprieties in an NlpE-dependent manner [49]. However, whether ZraSR responses to the envelope stress in the same manner needs further exploration.

The type 1 fimbrial adhesion, plays an important role in adherence to host cell to establish colonization in *E. coli*. In urinary tract infections (UTIs), UPEC uses the type 1 fimbriae to adhere to mannosylated glycoproteins on the surface of the urothelial epithelium [28,52]. This interaction not only facilitates initial attachment but also promotes the formation of intracellular bacterial communities (IBCs) and biofilms, enhancing UPEC’s ability to persist and cause disease [53]. The regulation of type 1 fimbriae expression is also complicated in UPEC. For instance, EnvZ/OmpR represses *fimB* transcription under acidic/hyperosmotic stress, which regulating type 1 fimbriae phase variation by changing the optimal expression of the FimB and FimE recombinases to reduce type 1 fimbriae [20]. GrpP/GrpQ, activated by urinary D-serine, binds directly to *fimS* to promote the phase-ON state and enhance type 1 fimbriae expression facilitating invasion to the bladder epithelial cells [36]. Moreover, previous study identified that CpxAR contributes to adherent and invasive *E. coli* (AIEC) gut competitive colonization by activating type 1 fimbriae expression in response to envelope stress [54]. Our work adds a novel dimension to this regulatory landscape: the ZraSR system is activated by a mechanical cue – host attachment – and directly biases the *fimS* element toward the phase-ON state without affecting *fimB* or *fimE* transcription (Figure 4). This establishes ZraSR as a unique TCS that co-opts a bet-hedging mechanism to translate a physical interaction into a precise virulence adaptation.

The invertible element *fimS* functions as an upstream genetic switch that governs the ON/OFF transcription of the entire *fim* operon (*fimAICDFGH*), thereby directly controlling *fimH* expression [18]. FimH acts as the central functional effector that directly mediates bacterial adhesion to host cells and subsequent invasion [14]. Phase switching of *fimS* directly determines the transcription level of *fimH*; locking *fimS* in the OFF state abolishes FimH production, while locking it ON restores the phenotype even in the Δ*zraSR* mutant. Indeed, genetically locking *fimS* in the ON orientation completely abolished the invasion defect of the Δ*zraSR* mutant, providing direct genetic proof that *fimS* orientation is the principal regulatory output of ZraSR in this pathway (Figure 3). Thus, *fimS* is the regulatory switch, and FimH is the decisive functional determinant for bacterial adhesion and invasion [16].

The upregulation of *fimH* by the ZraSR pathway ultimately targets the key interaction between FimH adhesin and its host receptor, CD48, on the surface of HBMECs (Figure 3). This FimH-CD48 binding is not merely for adhesion but acts as a critical trigger for host signaling cascades that promote bacterial internalization, via mechanisms such as elevated cytosolic calcium and Rho GTPase activation [55,56]. Our discovery that ZraSR activation upon host contact specifically enhances *fimH* transcription, which explains how the bacterium guarantees a high level of this crucial adhesin at the decisive moment of infection. Furthermore, the functional importance of this axis is confirmed by our data showing that CD48 knockdown reduces the invasion frequency of the wild-type strain (Figure 3). Therefore, the ZraSR-FimH-CD48 axis represents a coordinated virulence strategy: a mechanical attachment cue sensed by ZraSR leads to amplified *fimH* expression, thereby maximizing the FimH-CD48 signaling essential for efficient invasion and subsequent penetration of the BBB.

## Supplementary Material

Supplementary Data 1.xlsx

Clean Copy of Supplementary Material - QVIR-2026-0018.R1.docx

## Data Availability

RNA-seq data have been deposited in the NCBI SRA database under accession code PRJNA1392831. All raw data and supplementary materials supporting the findings of this study are openly available in figshare at https://doi.org/10.6084/m9.figshare.31054993 [57].
