# Supplementary material for: Identification of a mechanical signaling pathway activating type 1 fimbriae via a ZraSR two-component system in neonatal meningitis-associated Escherichia coli
Source: Virulence. 2026 Sep 11;17(1):2721762. doi: 10.1080/21505594.2026.2721762 (PMC13577287; doi:10.1080/21505594.2026.2721762)
Supplement: Supplementary file 1 [file KVIR_A_2721762_SM4346.docx]

**Supporting Information for**

**Identification of a mechanical signaling pathway activating type 1 fimbriae via a ZraSR two-component system in neonatal meningitis-associated *Escherichia coli***

Xiaoya Li^a,#^, Jiake Wang^a,#^, Zhengang Li^a,#^, Xi Guo ^a,b,#^, Yuchen Wang^a^, Xintong Chen^a^, Yangyang Zheng^c,d^, Qiyue Zhang^a^, Ruiying Liu^a^, Xuewei Sun^e^, Bin Liu^a,b,f,*^, Hao Sun^a,b,*^

^a^ National Key Laboratory of Intelligent Tracking and Forecasting for Infectious Diseases, TEDA Institute of Biological Sciences and Biotechnology, Nankai University, Tianjin 300457, China.

^b^ The Key Laboratory of Molecular Microbiology and Technology, Ministry of Education, Nankai University, Tianjin 300071, China.

^c^ State Key Laboratory of Experimental Hematology, National Clinical Research Center for Blood Diseases, Haihe Laboratory of Cell Ecosystem, Institute of Hematology & Blood Diseases Hospital, Chinese Academy of Medical Sciences & Peking Union Medical College, Tianjin 300020, China.

^d^ Tianjin Institutes of Health Science, Tianjin 301600, China.

^e^ Department of Critical Care Medicine, Children's Hospital of Tianjin University/Tianjin Children's Hospital, Tianjin, China.

^f^ Nankai International Advanced Research Institute, Shenzhen, China.

^#^ These authors contributed equally: Xiaoya Li, Jiake Wang, Zhengang Li and Xi Guo.

^*^ To whom correspondence may be addressed. E-mail: sunhao@nankai.edu.cn or [liubin1981@nankai.edu.cn](mailto:liubin1981@nankai.edu.cn).

**This PDF file includes:**

Supplementary Figures 1 to 5

Supplementary Tables 1 to 2

**Other supporting materials for this manuscript include the following:**

Supplementary Data 1 (separate file): Differentially expressed genes in *Escherichia coli* K1 WT and Δ*zraSR*.

**Supplementary Figures**

**Fig. S1 *zraSR* promotes the virulence of** *E. coli* K1**. (A)** Growth curves of *E. coli* K1 WT and Δ*zraSR* strains in LB medium. n = 3 independent experiments. **(B)** Growth curves of *E. coli* K1 WT and Δ*zraSR* strains in DMEM. n = 3 independent experiments. Two-way ANOVA **(A-B)** were applied.

**Fig. S2 ZraSR TCS positively regulates the type 1 fimbriae expression. (A)** qRT-PCR analysis of *glpK* and *secY* expression in WT and Δ*zraSR* strains in LB broth. n = 3 independent experiments. **(B-C)** Western blot analysis of FimH-3×FLAG expression in WT or *zraR*-D56A strains grown in LB broth **(B)** or during infection with HBMECs **(C)**. n = 3 independent experiments. DnaK was used as the loading control. Two-tailed Student’s *t* test **(A-C)** were applied.

**Fig. S3 ZraSR promotes *E.* *coli* K1 invasion via *fimH*.** Western blot analysis of control and CD48-knockdown HBMECs. n = 3 independent experiments. Hsp70 was used as the loading control.

**Fig. S4 ZraR interacts with *fimS* directly resulting in the bias towards the ON orientation.** Competitive EMSA of the *rpoS* DNA fragment with purified ZraR protein.

**Fig. S5** ZraSR is activated by a mechanical cue generated by initial host adherence**. (A)** qRT-PCR analysis of the *ntpA* or *gcvR* expression in *E. coli* K1 grown in LB broth and during HBMECs infection. n = 3 independent experiments. **(B)** qRT-PCR analysis of the *ntpA* or *gcvR* expression in *E. coli* K1 grown in LB broth and infection medium. n = 3 independent experiments. **(C)** qRT-PCR analysis of the *ntpA* or *gcvR* expression in *E. coli* K1 grown in LB broth, infection medium of non-infected HBMECs and infection medium of infected HBMECs. n = 3 independent experiments. **(D)** Representative images and quantification of the average GFP intensity per bacterium of WT-*fimS*-*gfp* and Δ*zraSR*-*fimS*-*gfp* reporter strains in uninfected versus infected fixed HBMECs. DNA was stained with DAPI and reporter activation was detected by GFP. Scale bar, 10 μm. The data are presented as mean ± SD. Two-tailed Student’s *t* test were applied.

**Supplementary Tables**

**Supplementary Table 1. Plasmids and strains used in this study.**

| **Plasmid** | **Genotype or description** | **Source** |
| --- | --- | --- |
| pKD3 | Template plasmid containing the Cm cassette for λ Red recombination; Cm^R^ | Datsenko KA, 2000 |
| pCP20 | FLP helper plasmid, temperature sensitive; Amp^R^ | W. Wackernagel, 1995 |
| pTrc99A | T7 expression vector; Amp^R^ | Amann E, 1988 |
| pET28a-*zraR* | pET28a carrying *zraR* gene; Km^R^ | This study |
| pETDute-*fimS*-*gfp* | pETDuet harboring *fimS-gfp* gene; Amp^R^ | This study |
| pETDute-P*zra*-*gfp* | pETDuet harboring P*zra-gfp* gene; Amp^R^ | This study |
| **Strains** | **Genotype or description** | **Source** |
| WT | Wild-type strain (*Escherichia* *coli* K1) | Lab collection |
| ∆*zraSR* | *Escherichia* *coli* K1 *zraSR*:: Cm; Cm^R^ | This study |
| ∆*fimH* | *Escherichia* *coli* K1 *fimH*:: Cm; Cm^R^ | This study |
| ∆*zraSR∆fimH* | ∆*zraSR* strain *fimH*:: Cm; Cm^R^ | This study |
| c*zraSR* | ∆*zraSR* containing plasmid pTrc99A with *zraSR*; Cm^R^, Amp^R^ | This study |
| FimH-FLAG | *Escherichia* *coli* K1 with the *fimH* gene with 3×FLAG tag; Cm^R^ | This study |
| WT-*fimS-gfp* | *Escherichia* *coli* K1 containing pETDuet harboring *fimS-gfp* gene; Amp^R^ | This study |
| ∆*zraSR*-*fimS-gfp* | ∆*zraSR* containing pETDuet harboring *fimS-gfp* gene; Amp^R^ | This study |
| WT-P*zra-gfp* | *Escherichia* *coli* K1 containing pETDuet harboring *zraSR* promoter-*gfp* gene; Amp^R^ | This study |

**Supplementary Table 2. Primers used in this study**

| **Primers** | **Sequence (5’-3’)** | **Experiment** |
| --- | --- | --- |
| *zraSR*-M-F | TTTCTGCTGTCAGAAAGGGATGAGCAGGCAAAGAAGAAGGTGTAGGCTGGAGCTGCTTC | Mutation construction |
| *zraSR*-M-R | CGCGACAGTTTTGCCAATAGCGTTTTGCGCGTGATCCCTCATATGAATATCCTCCTTAG |  |
| *zraSR*-I-F | CGCTGATATGACGGGTAATCTG | Identification |
| *zraSR*-I-R | ACTCCGTACTTCACCACCAA |  |
| c*zraSR*-F | CGGAATTCATGAGCAGGCAAAGAAGAAG | Complement construction |
| c*zraSR*-R | CCCAAGCTTGAGCGCCGCGCAGTTGAGCG |  |
| pTrC99a-I-F | TGGCTGTGCAGGTCGTAAATC | Complement  identification |
| pTrC99a-I-R | CCACACTACCATCGGCGCTA |  |
| *zraR*-RT-F | GGTGGTGGATGATGACATTAGC | qRT-PCR |
| *zraR*-RT-R | CCAGCGGCTTGATGAGATAATC |  |
| *zraS*-RT-F | GGTCCTGTTGAATCTCTATCT |  |
| *zraS*-RT-R | CTGTCGGTAACGCTGATT |  |
| *zraR*-*flag*-F | TGCTCTAGAATGCGTTTTATGCAACGTTC | Tag-flag  construction |
| *zraR*-*flag*-R | CCCAAGCTTTTACTATTTATCGTCGTCATCTTTGTAGTCGATATCATGATCTTTATAATCACCGTCATGGTCTTTGTAGTCACGCGACAGTTTTGCCAATAGCGTTTT |  |
| *zraR*-pET-F | CATGCCATGGCCATGCGTTTTATGCAACGTTCTAAAG | pET-ZraR |
| *zraR*-pET-R | CCCAAGCTTCCAGCGGTTTTCGCTACGTGCG |  |
| T7 | TAATACGACTCACTATAGGG | pET28a identification |
| T7/ter | TAATACGACTCACTATAGGG |  |
| *fimH*-M-F | ACCCAAAGAGATGATTGTAATGAAACGAGTTATTACCCTGTTTGCTGTACTGCTGATGGGTGTAGGCTGGAGCTGCTTCG | Mutation construction |
| *fimH*-M-R | TGCAGGTTTTAGCTTCAGGTAATATTGCGTACCTGCATTAGCAATGCCCTGTGATTTCTCATATGAATATCCTCCTTAG |  |
| *fimH*-I-F | TGGCAACACATTGAATACTGG | Identification |
| *fimH*-I-R | ACTCCGTACTTCACCACCAA |  |
| *fimH*-*flag*-F | AATGTGCAATCGATTATTGGCGTGACTTTTGTTTATCAAGACTACAAAGACCATGACGGTGATTATAAAGATCATGATATCGACTACAAAGATGACGACGATAAATAGTAAGTGTAGGCTGGAGCTGCTTCG | Tag-flag  construction |
| *fimH*-*flag*-R | AATATTGCGTACCTGCATTAGCAATGCCCTGTGATTTCTCATATGAATATCCTCCTTAG |  |
| *fimH-flag*-I-F | GGGCAACTCGATTTTCACC | Tag-flag identification |
| *fimH-flag*-I-R | GCAGGTTCAGACACATTCCCTA |  |
| *fim*ON-F | TAAAAAGAGAAGAAGCTTGATTTAACTAATTGATAAT AAAGTTAAAAAAGTGTAGGCTGGAGCTGCTTC | *fim*ON construction |
| *fim*ON-R | CGATGCTTTCCTCTATGAGTCAAAAGAGATCTAATTGTCTTGT ATTTATTTGCATATGAATATCCTCCTTAG |  |
| *fim*ON-I-F | GTAACGCAGACTCATCCTCATA | *fim*ON identification |
| *fim*ON-I-R | TACGAACCTGTCCTAACTGAAC |  |
| *fimA*-RT-F | CGTTCAGTTAGGACAGGTT | qRT-PCR |
| *fimA*-RT-R | ACAGTGGTATCGCAATCAT |  |
| *fimB*-RT-F | ACTGGAGATTCATCCGCACAT |  |
| *fimB*-RT-R | CTGTCGTCCTCTGGCTCTATC |  |
| *fimC*-RT-F | TACCTTGCGTATTCTTGATG |  |
| *fimC*-RT-R | TAATGCGGCTGATAATTGC |  |
| *fimD*-RT-F | GCTGGAAGACAACAACCT |  |
| *fimD*-RT-R | GCTATGGCTGTAACCGATA |  |
| *fimE*-RT-F | TGCGTATTAGTGAACTGCTTGA |  |
| *fimE*-RT-R | TATGAGGATGAGTCTGCGTTAC |  |
| *fimF*-RT-F | CGACGACTCCTGTTGTTC |  |
| *fimF*-RT-R | GGTTGGCATTGTGGCTAT |  |
| *fimG-*RT*-F* | CCACCAATGCCACGGTAGAT |  |
| *fimG-*RT*-R* | TGTTGCCACTGTCATCCTGTA |  |
| *fimH*-RT-F | GTGCCGATTCCTCTTACC |  |
| *fimH*-RT-R | CTACTGCTCCTAACGATACC |  |
| *fimI*-RT-F | TATTGGCGTAGCGTTGTT |  |
| *fimI*-RT-R | TTTGGCGATGAAATGTAGC |  |
| *fimS*-RT-F | CAGTAATGCTGCTCGTTTTGCCG |  |
| *fimS*-RT-R | CAGAGCCGACAGAACAAC |  |
| *rpoS*-RT-F | TTGATGAGAACGGAGTAGAG |  |
| *rpoS*-RT-R | TTAGCAGAGGCGAATAACC |  |
| *rpoA*-RT-F | GTGACCCTTGAGCCTTTAGAG |  |
| *rpoA*-RT-R | ACACCATCAATCTCAACCTCG |  |
| *ntpA*-RT-F | GCCACTTGTAAGCCAGAT |  |
| *ntpA*-RT-R | AAGGAAGAGGTCACCATTG |  |
| *gcvR*-RT-F | ACACTGTCATCGCAACAT |  |
| *gcvR*-RT-R | CAATCAGAGTAATGGCATTCC |  |
| *glpK*-RT-F | TTACTCGTCGTGTTCTTCC |  |
| *glpK*-RT-R | GCCTATCTTGCTGGTCTG |  |
| *secY*-RT-F | AGCGGTGAAGTAGAATGC |  |
| *secY*-RT-R | GAGTCTGGTCGTCGTAAG |  |
| *fimS*-F | TTGGGGCCAAACTGTCTATA | *fimS* for EMSA |
| *fimS*-R | TTGGGGCCATTTTGACTCAT |  |
| *fimS* 601-F | CAGTAATGCTGCTCGTTTTGCCG | Cloning of *fimS* gene |
| *fimS* 601-R | CAGAGCCGACAGAACAAC |  |
| pETDuet-I-F | TAATACGACTCACTATAGGG | pETDuet identification |
| pETDuet-I-R | TGCTAGTTATTGCTCAGCGG |  |
| *zra*-*pro*-F | CATCTTCCGTGCCAACGGCGAAACGCTG | promoter of *zra* |
| *zra*-*pro*-R | CTTCTTCTTTGCCTGCTCATCC |  |
| pETDuet-P*zra*-*gfp*-F1 | CGGAATTCTATTGCATCTTCCGTGCCAA | Cloning of *zra-gfp* gene to pETDuet plasmid |
| pETDuet-P*zra*-*gfp*-R1 | ATGAGTAAAGGAGAAGAACTTTTCACTGGAGTTGTCCCCTTCTTCTTTGCCTGCTCAT |  |
| pETDuet-P*zra*-*gfp*-F2 | ATGAGCAGGCAAAGAAGAAGGGGACAACTCCAGTGAAAAGTTCTTCTCCTTTACTCAT |  |
| pETDuet-P*zra*-*gfp*-R2 | CCCAAGCTTCTATTTGTATAGTTCATCCATGCCATGTGTAATCCCAGC |  |
| pETDuet-*fimS-gfp*-F1 | CGGAATTCTTGGGGCCATTTTGACTCAT | Cloning of *fimS-gfp* gene to pETDuet plasmid |
| pETDuet-*fimS-gfp*-R1 | CCAGTGAAAAGTTCTTCTCCTTTACTCATTTGGGGCCAAACTGTCTATA |  |
| pETDuet-*fimS-gfp*-F2 | TATAGACAGTTTGGCCCCAAATGAGTAAAGGAGAAGAACTTTTCACTGG |  |
| pETDuet-*fimS-gfp*-R2 | AACTGCAGTTATTTGTATAGTTCATCCATGCCATGTGTAATC |  |
